# Involvement of CYP2E1 in the Course of Brain Edema Induced by Subacute Poisoning With 1,2-Dichloroethane in Mice

**DOI:** 10.3389/fphar.2018.01317

**Published:** 2018-11-15

**Authors:** Xiaoxia Jin, Yingjun Liao, Xiaoqiong Tan, Gaoyang Wang, Fenghong Zhao, Yaping Jin

**Affiliations:** ^1^Department of Environmental and Occupational Health, School of Public Health, China Medical University, Shenyang, China; ^2^Department of Physiology, China Medical University, Shenyang, China

**Keywords:** 1, 2-dichloroethane (1, 2-DCE), cytochrome P450 2E1 (CYP2E1), brain edema, diallyl sulfide (DAS), nuclear factor erythroid 2-related factor 2 (Nrf2), heme oxygenase-1 (HO-1)

## Abstract

This study was designed to explore the role of cytochrome P4502E1 (CYP2E1) expression in the course of brain edema induced by subacute poisoning with 1,2-dichloroethane (1,2-DCE). Mice were randomly divided into five groups: the control group, the 1,2-DCE poisoned group, and the low-, medium- and high-dose diallyl sulfide (DAS) intervention groups. The present study found that CYP2E1 expression levels in the brains of the 1,2-DCE-poisoned group were upregulated transcriptionally; in contrast, the levels were suppressed by DAS pretreatment in the intervention groups. In addition, the expression levels of both Nrf2 and HO-1 were also upregulated transcriptionally in the brains of the 1,2-DCE-poisoned group, while they were suppressed dose-dependently in the intervention groups. Moreover, compared with the control group, MDA levels and water contents in the brains of the 1,2-DCE-poisoned group increased, whereas NPSH levels and tight junction (TJ) protein levels decreased significantly. Conversely, compared with the 1,2-DCE- poisoned group, MDA levels and water contents in the brains of the intervention groups decreased, and NPSH levels and TJ protein levels increased significantly. Furthermore, pathological changes of brain edema observed in the 1,2-DCE-poisoned group were markedly improved in the intervention groups. Collectively, our results suggested that CYP2E1 expression could be transcriptionally upregulated in 1,2-DCE-poisoned mice, which might enhance 1,2-DCE metabolism *in vivo*, and induce oxidative damage and TJ disruption in the brain, ultimately leading to brain edema.

## Introduction

The synthetic halocarbon 1,2-dichloroethane (1,2-DCE, CAS number: 107-06-2) is widely manufactured around the world and is used primarily in the production of vinyl chloride (Yu et al., 2013; Sun et al., 2016). 1,2-DCE is a colorless and volatile organic liquid; thus, it is also used as a general organic solvent in China and other countries. It can be evaporated quickly into the air when 1,2-DCE is used as a thinner of industrial adhesives, as a consequence, workers may inhale high vapor concentrations of 1,2-DCE in the workplace. Accumulated evidence has found that exposure to high concentrations of 1,2-DCE through the air could cause acute and subacute poisoning in both workers and laboratory animals (Hotchkiss et al., 2010; Wang et al., 2014; Zhou et al., 2015). Based on data reported in China, many occupational poisoning accidents induced by 1,2-DCE have occurred in the past 30 years (Liu et al., 2010). Toxic encephalopathy is the typical symptom, and brain edema is the main pathological change (Yang et al., 2009; Wang et al., 2013; Chen S. et al., 2015). Therefore, subacute poisoning of 1,2-DCE is one of the most serious occupational problems in current China. However, our understanding in relation to the mechanisms of 1,2-DCE-induced brain edema is limited.

Considerable evidence has indicated that 1,2-DCE is metabolized via cytochrome P450 2E1 (CYP2E1) in the body (Guengerich et al., 1980; Sweeney et al., 2008; Sun et al., 2016). To date, studies have found that the metabolites, i.e., 2-chloroethanol and chloroacetaldehyde, formed by CYP2E1-mediated metabolism are more reactive than their parent compound (Raucy et al., 1993; Gonzalez, 2005). The CYP2E1-mediated metabolism of 1,2-DCE has been proposed to play an important role in the mechanism underlying 1,2-DCE-induced toxicity (Storer and Conolly, 1985). Furthermore, compared with the other forms of CYP450s, more reactive oxygen species (ROS) could be generated in CYP2E1-mediated metabolism due to the intense activity of NADPH oxidase (Liu et al., 2005). Thus, oxidative damage might occur during the metabolic process. More importantly, CYP2E1 expression could be upregulated by substrates for the enzyme in both humans and animals (Das et al., 2010; Zhang et al., 2015). Accordingly, the *in vivo* toxicities of such compounds could be further enhanced due to the upregulated expression of CYP2E1 (Wargovich, 2006).

Although CYP450s are most abundant in the liver, they are also expressed in the extrahepatic tissues (Garcia-Suastegui et al., 2017). Previous studies have found that CYP2E1 is present constitutively in both glial and neuronal cells of rat brain (Vichi et al., 2015). In the brain, it has been reported that the basal ganglia, the frontal cortex and the hippocampus have high density levels of CYP2E1 expression; morphological and biochemical changes were also focused in these regions due to ethanol consumption in the experimental animals (Ledesma et al., 2014; Toselli et al., 2015). Because CYP2E1 is the main catalyst of ethanol in the brain, the correlation between the presence of CYP2E1 and the toxic effects of ethanol in the same brain regions suggested that CYP2E1-mediated alcohol metabolism in the brain might play an important role in alcohol neurotoxicity. Thus, it was reasonable to hypothesize that upregulated expression of CYP2E1 in the brain might be implicated in the brain edema formation induced by subacute poisoning with 1,2-DCE in mice, and most likely also in human beings.

As a potent inhibitor of CYP2E1, diallyl sulfide (DAS) has received much more attention in the protection against the toxicities of exogenous chemicals due to the suppressed expression of CYP2E1 (Yang et al., 2001; Davenport and Wargovich, 2005; Zhai et al., 2008). A recent study demonstrated that treatment of mice with 1,2-DCE could enhance CYP2E1 protein expression and enzymatic activity and cause oxidative damage in the liver, which could be attenuated by DAS pretreatment, suggesting that CYP2E1 plays an important role in 1,2-DCE-induced liver damage (Sun et al., 2016). However, is this enzyme also implicated in the brain edema formation induced by subacute poisoning with 1,2-DCE? To date, no study has been designed to investigate this issue. Resolving this issue would considerably advance our understanding of the mechanisms underlying 1,2-DCE-induced brain edema and could impact the policies and clinical practices concerning the prevention and treatment of 1,2-DCE poisoning.

## Materials and Methods

### Animal Care and Use Statement

This study was conducted in accordance with the National Institutes of Health guidelines in a manner that minimized animal suffering and animal numbers and has been approved by the Scientific Research Committee of China Medical University on Ethics in the Care and Use of Laboratory Animals.

### Animals

Female Kunming albino mice used in this study were obtained from the Experimental Animal Center of China Medical University. The animal room was kept at a temperature of 22–24°C with a 12 h light/dark cycle and a relative humidity of 50–60%. Mice weighing between 23 and 26 g were housed five per cage in sterilized plastic cages with wood shaving bedding. Food and water were available freely to the animals. During the course of the study, mice were weighed and observed carefully for the symptoms of poisoning every day.

### Groups

#### Roles of CYP2E1 in 1,2-DCE Metabolism

After 1-week adaptation, ten mice were randomly divided into two groups: the 1,2-DCE-poisoned group and the DAS intervention group, with five mice in each group. Mice in the poisoned group were treated by gavage with 0.2 ml corn oil every day, while those in the intervention group were treated by gavage with 50 mg/kg body weight (b.w) DAS dissolved in 0.2 ml corn oil. Four hours after intragastric administration, the mice in both groups were exposed to 1.2 g/m^3^ 1,2-DCE (initial concentration), 3.5 h per day for 3 days. Following every exposure, the mice from both groups were placed separately in metabolic cages for 4 h, and their urinary samples were collected every day.

#### Roles of CYP2E1 in 1,2-DCE-Induced Brain Edema

Fifty mice were randomly divided into five groups: the control group, the 1,2-DCE-poisoned group (poisoned group), and the low-, medium-, and high-dose DAS intervention groups (intervention group), with ten mice in each group. Mice in the control and poisoned groups were treated by gavage with 0.2 ml corn oil, and those in the intervention groups were treated by gavage with 25, 50, and 100 mg/kg b.w DAS dissolved in 0.2 ml corn oil, respectively. Four hours later, mice in both the poisoned group and the intervention groups were exposed to 1.2 g/m^3^ 1,2-DCE, 3.5 h per day, for 3 days. Mice in the control group were kept in the chamber without exposure to 1,2-DCE. One day after the last exposure, all mice from each group were deeply anesthetized and sacrificed by decapitation. Their brains were removed immediately for further analysis.

### Treatment

Static inhalation exposure was used in this study for 1,2-DCE exposure. Mice from different groups were placed in an exposure chamber with a capacity of 100 L, with five mice in each chamber. 1,2-DCE with a purity of more than 99% was weighed and placed on a plate suspended in the chamber and was then evaporated by a fan in the chamber after it was sealed. The administered concentration of 1,2-DCE was calculated by the weight divided by the chamber volume. At the end of the exposure, the mice were removed from the inhalation chambers immediately.

The benefit of static inhalation exposure is that it is inexpensive, easy to build and operate, and results in the consumption of less test chemicals. This method is particularly suitable for small animals in the experiment evaluating acute and subacute inhalation exposure (Wang et al., 2014). Data from the U.S. EPA (1998) and the Chinese Textbook of Toxicology indicated that up to ten mice are permitted in a 100 L exposure chamber for 4 h.

However, in this study, only five mice were kept in the exposure chamber and for 3.5 h. Data examined in our laboratory showed that at the end of exposure, the oxygen levels were close to 20%, the concentrations of carbon dioxide were lower than 2%, and the humidity was lower than 70% in the chamber. The time-weighted average concentration of 1,2-DCE in the chamber during the exposure course was 1.03 g/m^3^.

During the experimental period, mice were observed for poisoning symptoms and mortality and were weighed daily before exposure to 1,2-DCE. Body tremors and forelimb flexure could be observed in the mice exposed to 1,2-DCE for 2 and 3 days. The mortality rates of mice exposed to 1,2-DCE for 3 days were nearly 25%. However, in the present study, only surviving mice were used for further analysis. No mice in the control or intervention groups died during the experimental period.

### Reagents and Laboratory Wares

Antibodies against CYP2E1 (#AB1252), occludin (#40-4700), heme oxygenase-1 (HO-1, A1346) and β-actin were purchased from Millipore (United States), Thermo Fisher Scientific (United States), ABclonal (China), and Santa Cruz Biotech (United States), respectively. Antibodies against claudin-5 (#ab15106) and nuclear factor erythroid 2-related factor 2 (Nrf2, #ab62352) were the products of Abcam (Cambridge, MA, United States). Horseradish peroxidase was obtained from ZSGB Biotechnology (China). The assay kits for detecting superoxide dismutase (SOD) activity and the levels of malondialdehyde (MDA), non-protein sulfhydryl (NPSH) and creatinine were purchased from Nanjing Jiancheng Bioengineering Co. Ltd (China). DAS was the product of Tokyo Chemical Industry (Japan). A bicinchoninic acid (BCA) protein assay kit and an enhanced chemiluminescence (ECL) plus kit were the products of Thermo Fisher Scientific (United States). RIPA Lysis Buffer was the products of Beyotime Biotechnology (China). TRIzol reagent was purchased from Takara (Japan). All other chemicals used were analytical grade and were purchased from local chemical suppliers. Double-distilled water was used in this study.

### Analysis Process

#### Brain Water Content

Briefly, the right cerebral hemispheres of mice were dissected and weighed immediately on an analytical balance, with the weights represented as wet weight. The dry weight was obtained after drying the tissue in an oven at 100°C for 24 h. The brain water content was calculated as [(wet weight – dry weight)/wet weight] × 100%.

#### Histopathological Observation

Briefly, the left cerebral hemispheres of mice were removed and fixed immediately with 4% paraformaldehyde for 72 h, and embedded in paraffin. Serial coronal sections (5 μm) were collected and stained with hematoxylin and eosin (HE).

#### Western Blot Analysis

Briefly, cerebral samples were taken and homogenized. The cerebral homogenate was lysed in RIPA lysis buffer and then centrifuged at 12,000 × *g* for 20 min at 4°C. Protein concentrations in the prepared solution were measured with a BCA protein assay kit. Equal amounts of total protein from the cerebral samples were resolved by 8% SDS-PAGE and were then transferred and blotted onto polyvinylidene difluoride membranes (Millipore, United States), which were blocked with 5% skimmed milk at room temperature for 1.5 h. The target proteins on the membranes were probed with rabbit antibodies against CYP2E1 (1:2000), Nrf2 (1:1000), HO-1 (1:1000), claudin-5 (1:1000), ZO-1 (1:1000), occludin (1:500) and β-actin (1:2000) at 4°C overnight. The bands on the membranes were incubated with secondary antibody (1:5000) conjugated with horseradish peroxidase at room temperature for 1 h. The immunoreactivities of the bands were detected with an ECL plus kit. The band intensities were semiquantitatively assessed by densitometry using image analysis software (Gel-Pro analyzer v4.0) and were normalized to β-actin, which served as the internal control. The results are expressed as the relative intensity of target protein in the cerebral tissues.

#### Quantitative Real-Time RT-PCR Assay

Total RNA in the cerebral tissues was extracted with TRIzol Reagent. First-strand cDNA was synthesized using a Prime Script RT reagent kit (Takara, Japan) and was then amplified using SYBR Premix ExTaq II (Takara, Tokyo, Japan) and an ABI 7500 Real-Time PCR System (Applied Biosystems, United States). To amplify the fragments of CYP2E1, Nrf2, HO-1, claudin-5, ZO-1 and GAPDH, the primer pairs detailed in Table 1 were used. Amplification was conducted for 40 cycles of 5 s at 95°C and 34 s at 60°C. The results were evaluated using the comparative Ct method (Livak and Schmittgen, 2001). RNA abundance were expressed as 2^-ΔΔCt^ for the target mRNA normalized to the GAPDH gene (used as an internal control) and was presented as fold change vs. contralateral control samples.

**Table 1:** The sequence of primer pairs for PCR analysis.

Gene	Primer(5′→3′)	Primer sequences	Length(bp)
CYP2E1	Sense	CAGGAGTACAAGAACAAGGGG	195
	Antisense	CAGAAAGGTAGGGTCAAAAGG	
Nrf2	Sense	TTGGCAGAGACATTCCCATTTG	172
	Antisense	AAACTTGCTCCATGTCCTGCTCTA	
HO-1	Sense	TGCAGGTGATGCTGACAGAGG	144
	Antisense	GGGATGAGCTAGTGCTGATCTGG	
GAPDH	Sense	CAATGTGTCCGTCGTGGATCT	124
	Antisense	GTCCTCAGTGTAGCCCAAGATG	


#### SOD Activity and ROS and MDA Levels in the Brain

Briefly, brain homogenate was prepared and then centrifuged at 4°C, 12,000 *g* for 20 min. The supernatant was collected for analysis of MDA levels, SOD activity and NPSH contents using specific commercial kits. Protein concentrations in the homogenate were evaluated using BCA protein assay kits. The absorbance of the color formed was determined using a microplate reader (BioTek Instruments, United States).

#### Levels of Urinary Chloroacetic Acid

Briefly, urine samples were incubated with sulfuric acid and methanol at 60°C for 60 min. Then, *n*-hexane was added and mixed with a vortexer. After centrifugation, the supernatant was collected and detected using a gas chromatography (Agilent, model 7890A), equipped with an electron-capture detector and DB1701 GC column. The operating conditions were as follows: injector temperature, 250°C; column temperature, 180°C; detector temperature, 300°C; nitrogen flow rate, 30 ml/min; ECD range, 10; ECD attenuation, 4; chart speed, 0.5 cm/min; split ratio was 20:1; column flow, constant current mode; carrier gas, Ar with 5% CH_4_; velocity of flow, 1 ml/min. The volume injected was 1 μl. The oven conditions were as follows: an initial temperature of 35°C for 5 min, a ramp of 20°C/min from 35 to 70°C, keep 2 min; a ramp of 20°C/min from 70 to 200°C, keep 2 min; a ramp of 10°C/min from 200 to 220°C, keep 5 min. The average retention time of chloroacetic acid is 11.7 min.

A standard solution was prepared by the addition of chloroacetic acid to the pooled urine samples taken from mice without 1,2-DCE exposure. Levels of urinary creatinine were determined using a specific assay kit.

### Statistical Analysis

The group means and standard deviations of normally distributed data were calculated and analyzed by one-way analysis of variance (one-way ANOVA) using SPSS for Windows, version 18.0 (SPSS Inc. Chicago, IL, United States). The *post hoc* tests were performed using the Student–Newman–Keuls test (SNK). Levels of urinary chloroacetic acid were transformed logarithmically and expressed as geometric means and deviations, which were analyzed using the Student *t*-test. Statistical significance was assumed at *P* < 0.05.

## Results

### General Health of Mice During the Experimental Period

**FIGURE 1:**
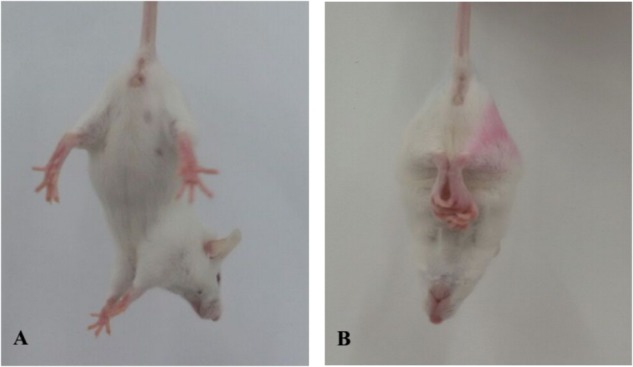
Comparison of limb flexure in mice between the control group (**A**, left) and the 1,2-DCE-poisoned group (**B**, right).

**FIGURE 2:**
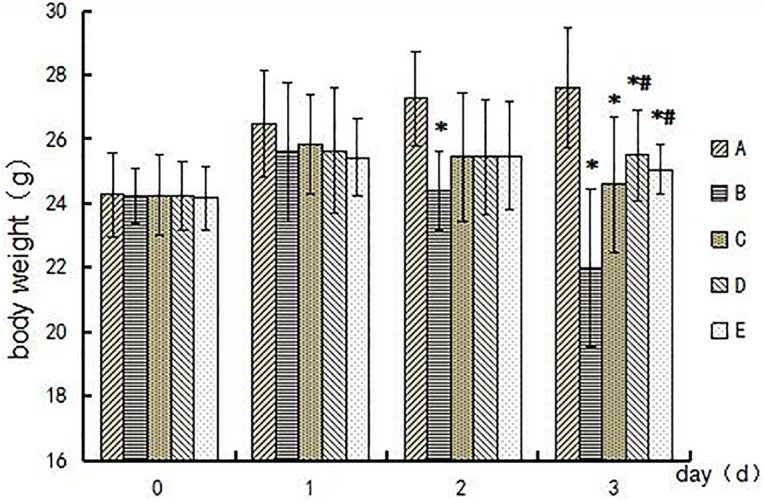
Comparison of mouse body weights among groups during the experimental period. Groups A to E represent the control group, the 1,2-DCE-poisoned group, and the low-, medium-, and high-dose DAS intervention groups, respectively. Data are expressed as the means ± SD and were analyzed by one-way ANOVA. Significant difference was defined as p less than 0.05, and, ^∗^, vs. the control group; **^#^**, vs. the 1,2-DCE-poisoned group. The number of mice in each group was 5.

Symptoms of body tremor and forelimb flexure in mice in the poisoned group could be observed after 2 days of exposure and became more severe at the end of 3 days of exposure (Figure 1). However, there were no poisoning symptoms as aforementioned in the control group, and no symptoms of forelimb flexure were observed in the intervention groups. The body weights of mice in the poisoned group and the intervention groups decreased significantly at the end of the 3-day exposure compared with the control group. In contrast, the weights of the mice in the medium- and high-dose intervention groups were significantly higher than those in the poisoned group (Figure 2).

### Effects of DAS on 1,2-DCE Metabolism in the Body

To evaluate the CYP2E1-mediated metabolism of 1,2-DCE in mice, the urine samples of mice were collected daily at the end of exposure, and levels of chloroacetic acid, the final metabolite of 1,2-DCE *in vivo*, were determined. As shown in Table 2, the urinary levels of chloroacetic acid in the poisoned group increased significantly over the exposure days. In contrast, the urinary levels in the intervention group did not differ significantly over the exposure days. Furthermore, those in the poisoned group were significantly higher than those in the intervention group from the second exposure day.

**Table 2:** Comparison of chloroacetic acid level in the urine of mice among groups.

Group	1d	2d	3d
1,2-DCE-poisoned group	10.96 ± 2.88^(∗)^	100.00 ± 1.32^#(∗)^	281.84 ± 1.32^#Δ(∗)^
DAS-intervention group	3.16 ± 1.86	3.09 ± 1.26^∗^	1.35 ± 2.75^∗^


*Data are presented as geometric means ± geometric SD (mg/g creatinine). They were analyzed using the Student independent-sample *t*-test between the 1,2-DCE-poisoned group and the DAS intervention group on the same exposure day and using one-way ANOVA among different exposure days within the same group. Significant differences were defined as *p-*values less than 0.05. ^∗^, vs. the 1,2-DCE-poisoned group; ^#^, vs. the first day in the 1,2-DCE-poisoned group; ^Δ^, vs. the second day in the 1,2-DCE-poisoned group. The number of mice in each group was 5.*

### Effects of DAS on CYP2E1 Expression in the Brain

**FIGURE 3:**
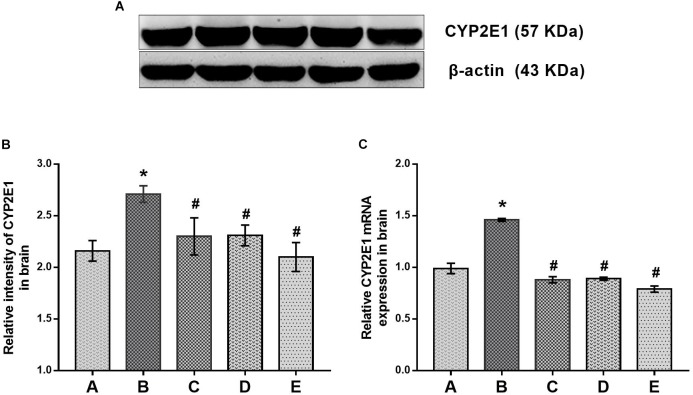
Comparisons of CYP2E1 protein and mRNA expression levels in the brains of mice among the groups. **(A)** Western blot analysis. Images are the representative results of five separate experiments for each group. **(B)** Densitometric analysis of Western blots, showing the relative intensity in arbitrary units compared with β-actin. **(C)** Quantitation of mRNA by real-time RT-PCR. Gene expression was normalized to GAPDH and is presented as fold change vs. the control group. Groups A to E represent the control group, the 1,2-DCE-poisoned group, and the low-, medium-, and high-dose DAS intervention groups, respectively. Data are expressed as the means ± SD and were analyzed by one-way ANOVA. Significant difference was defined as p less than 0.05. ^∗^, vs. group control group; ^#^, vs. group 1,2-DCE-poisoned group. The number of mice in each group was 5.

Evidence for the effects of DAS on CYP2E1 expression in the brain is shown in Figure 3. The protein and mRNA levels of CYP2E1 in the brain of mice in the poisoned group increased significantly compared with the control group. Moreover, both the protein and mRNA levels of CYP2E1 in the brains of mice in the intervention groups decreased significantly compared with the poisoned group.

### Preventive Effects of DAS on Oxidative Damage in the Brain

**FIGURE 4:**
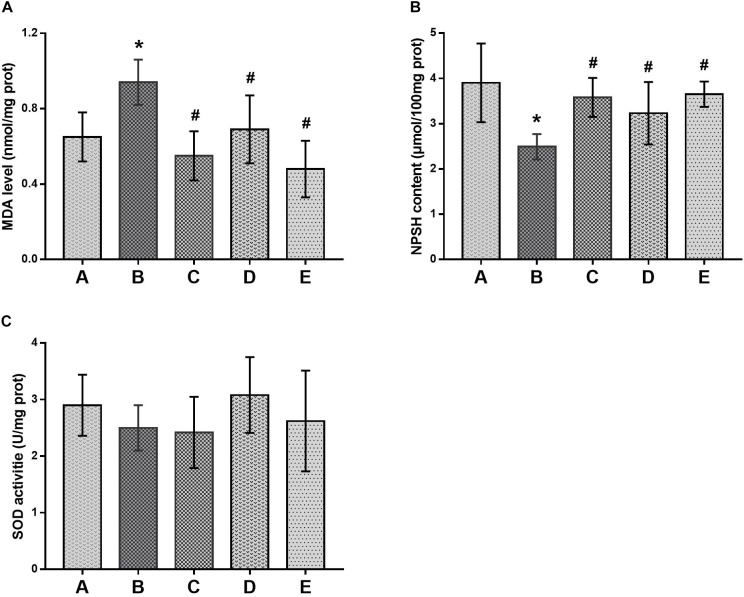
Comparison of MDA levels, NPSH contents and SOD activities in the brain of mice among groups. **(A)** Levels of MDA, **(B)** activity of SOD and **(C)** contents of NPSH were illustrated, respectively. Notes: NPSH, non-protein sulfhydryl; MDA, malondialdehyde; SOD, superoxide dismutase. Groups A to E represent the control group, the 1,2-DCE-poisoned group, and the low-, medium- and high-dose DAS intervention groups, respectively. Data are expressed as the means ± SD and were analyzed by one-way ANOVA. Significant difference was defined as p less than 0.05. ^∗^, vs. the control group; ^#^, vs. the 1,2-DCE-poisoned group. The number of mice in each group was 5.

Oxidative damage in the brain was evaluated by production of MDA and depletion of NPSH in the brains of mice, as shown in Figure 4. Compared with the control group, MDA levels in the brains of mice in the poisoned group increased, whereas the NPSH contents decreased significantly. In contrast, MDA levels in the brains of mice in the intervention groups decreased, but NPSH contents increased significantly compared with the poisoned group. However, they did not differ significantly among the intervention groups. In addition, SOD activities in the brains of mice did not differ significantly between the control and poisoned groups.

### Effects of DAS on Nrf2 and HO-1 Expression

**FIGURE 5:**
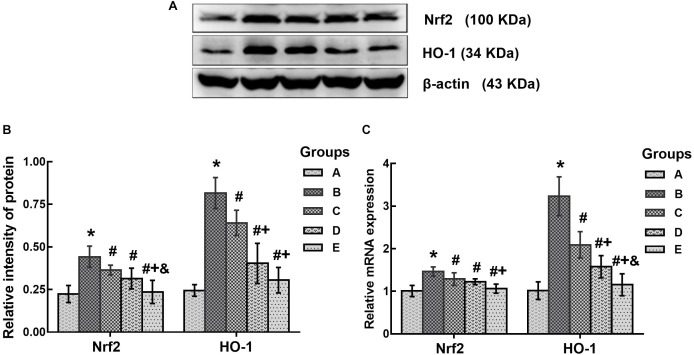
Comparison of Nrf2 and HO-1 protein and mRNA levels in the brains of mice among the groups. **(A)** Western blot analysis. Images are the representative results of five separate experiments for each group. **(B)** Densitometric analysis of Western blots, showing the relative intensity in arbitrary units compared with β-actin. **(C)** Quantitation of mRNA by real-time RT-PCR. Gene expression was normalized to GAPDH and is presented as fold change vs. the control group. Groups A to E represent the control group, the 1,2-DCE-poisoned group, and the low-, medium- and high-dose DAS intervention groups, respectively. Data are expressed as the means ± SD and were analyzed by one-way ANOVA. Significant difference was defined as p less than 0.05. ^∗^, vs. the control group; #, vs. the 1,2-DCE-poisoned group; **^+^**, vs. the low-dose DAS intervention group; **^&^**, vs. the medium-dose DAS intervention group. The number of mice in each group was 5.

In this study, Nrf2 and HO-1 expression levels were determined to evaluate the changes of oxidative stress in the brains of mice affected by treatment with 1,2-DCE or DAS. As shown in Figure 5, both the protein and mRNA levels of Nrf2 in the poisoned group increased significantly compared with the control group, whereas those in the intervention groups significantly decreased compared with the poisoned group. Moreover, those in the high-dose intervention group decreased significantly compared with the low-dose intervention group, and the protein levels of Nrf2 in the high-dose intervention group further decreased compared with the medium-dose intervention group. Likewise, both the protein and mRNA levels of HO-1 in the poisoned group increased significantly compared with the control group, while those in the intervention groups decreased significantly compared with the poisoned group. In addition, the mRNA levels of HO-1 in the intervention groups decreased significantly and dose-dependently, and the protein levels in the medium- and high-dose intervention groups decreased significantly compared with the low-dose intervention group.

### Preventive Effects of DAS on Brain Edema Formation

**FIGURE 6:**
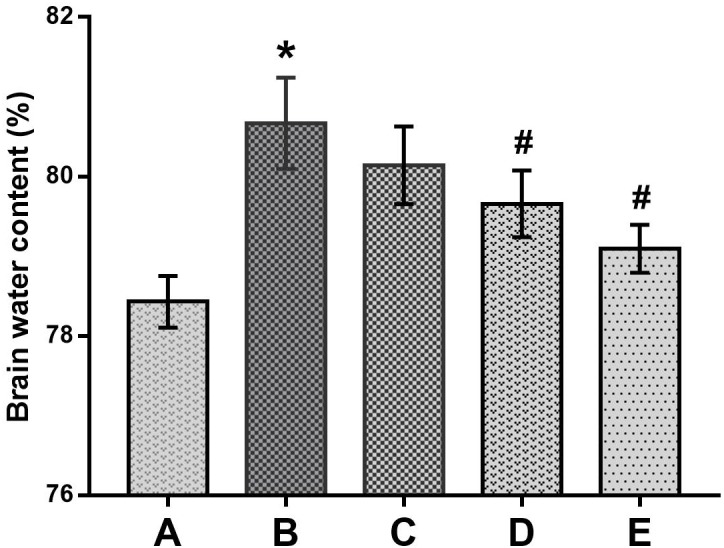
Comparison of brain water contents among the groups. Notes: Groups A to E represent the control group, the 1,2-DCE-poisoned group, and the low-, medium- and high-dose DAS intervention groups, respectively. Data are expressed as the means ± SD and were analyzed by one-way ANOVA. Significant difference was defined as *p* less than 0.05. ^∗^, vs. the control group; ^#^, vs. the 1,2-DCE-poisoned group. The number of mice in each group was 5.

In the present study, brain edema formation was evaluated according to the changes in histopathological observations and water contents in the brains of mice. As shown in Figure 6, the brain water contents in the poisoned group increased significantly compared with the control group. In contrast, those in the medium- and high-dose intervention groups decreased significantly compared with the poisoned group.

**FIGURE 7:**
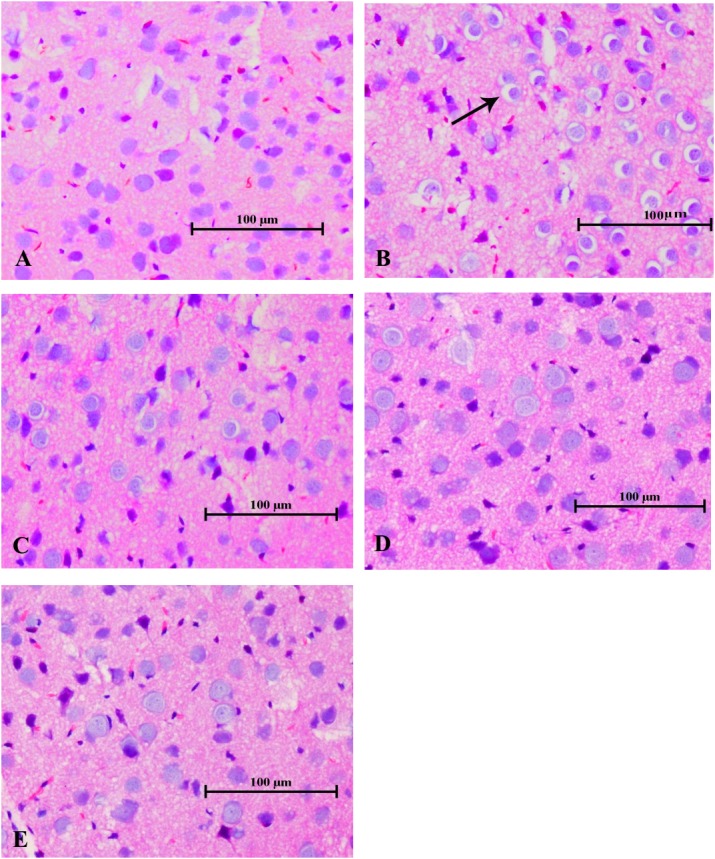
Changes in histological observations in the cerebral tissues of mice induced by 1,2-DCE poisoning (HE staining). The photomicrographs shown of HE staining **(A–E)** are representative of five separate experiments and were captured with an Olympus light microscope (200×). The tissue samples were stemmed from the parietal cortex. Groups A to E represent the control group, the 1,2-DCE-poisoned group, and the low-, medium-, and high-dose DAS intervention groups, respectively. Arrows indicate the enlarged perinuclear spaces.

As illustrated in Figure 7, the histopathological changes, with enlarged perinuclear spaces, widened lacunar spaces surrounding vessels, lightly stained intercellular matrix and cytoplasm, and swelling cell bodies in the cerebral tissues indicated the typical morphological characteristics of brain edema in the mice of the poisoned group. In contrast, only slight pathological changes were observed in the brains of mice in the intervention groups, such as enlarged perinuclear spaces in the low-dose intervention group, and lightly stained intercellular matrix and cytoplasm in the medium- and high-dose intervention groups. No pathological changes were observed in the cerebral tissues of the control mice.

### Effects of DAS on Expression of Tight Junction Proteins

**FIGURE 8:**
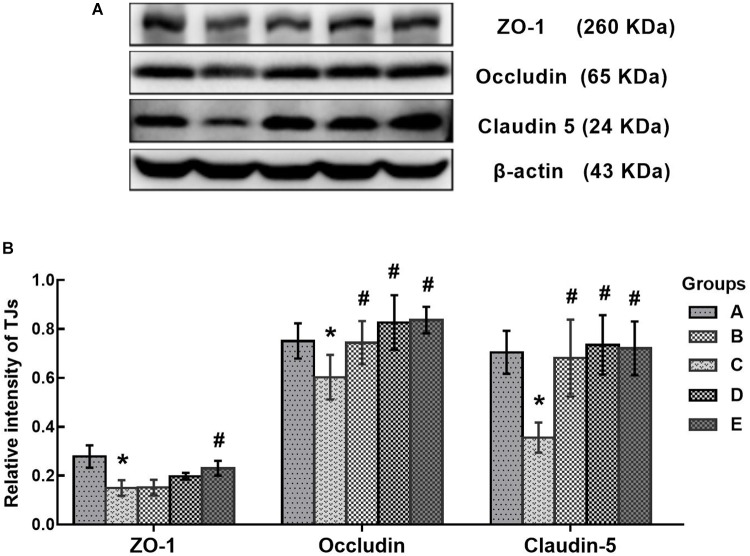
Comparison of TJs protein levels in the brains of mice among the groups. **(A)** Western blot analysis. Images are the representative results of five separate experiments for each group. **(B)** Densitometric analysis of Western blots, showing the relative intensity in arbitrary units compared with β-actin. Groups A to E represent the control group, the 1,2-DCE-poisoned group, and the low-, medium-, and high-dose DAS intervention groups, respectively. Data are expressed as the means ± SD and were analyzed by one-way ANOVA. Significant difference was defined as p less than 0.05. ^∗^, vs. the control group; #, vs. the 1,2-DCE-poisoned group. The number of mice in each group was 5.

In this study, disruption of the blood-brain barrier (BBB) integrity was assessed according to the expression of tight junction proteins in the brain. As shown in Figure 8, the protein levels of ZO-1, occludin and claudin-5 in the poisoned group decreased significantly compared with the control group. Conversely, the expression levels of ZO-1 in the high-dose intervention group, as well as occludin and claudin-5 in all intervention groups increased significantly compared with the poisoned group.

## Discussion

The present study found that CYP2E1 expression in the brains of 1,2-DCE-poisoned mice was upregulated transcriptionally and that pretreatment of 1,2-DCE-poisoned mice with DAS could efficiently suppress the enhanced expression of CYP2E1. In our recently published paper, we found that the protein levels and enzymatic activity of CYP2E1 in the livers of 1,2-DCE-poisoned mice were upregulated (Sun et al., 2016). However, to the best of our knowledge, this is the first report on CYP2E1 expression in the brains of 1,2-DCE-poisoned mice. Moreover, the above results were also supported by the data on urinary chloroacetic acid in 1,2-DCE-poisoned mice. It has been well established that 1,2-DCE *in vivo* can be dehalogenated and then oxidized to yield chloroacetic acid, which may appear in the urine as the final metabolite of 1,2-DCE. Therefore, levels of chloroacetic acid in the urine might reflect the amount of 1,2-DCE metabolized by CYP2E1 in mice. It has been reported that the concentration of 1,2-DCE in the blood of mice decreased dramatically after exposure; the half-life of 1,2-DCE in mice is within 1 h (Yang et al., 2016). Therefore, increased urinary levels of chloroacetic acid in 1,2-DCE-poisoned mice cannot be attributed to the accumulation of inhaled 1,2-DCE; instead, they must result from the potentiated metabolism of inhaled 1,2-DCE due to overexpression of CYP2E1. Furthermore, since the expression of CYP2E1 in 1,2-DCE-poisoned mice was suppressed efficiently by DAS pretreatment, the amount of CYP2E1-mediated 1,2-DCE metabolism *in vivo* failed to increase along with the exposure days. Thus, the urinary levels of chloroacetic acid in the intervention group did not increase along with the exposure days. Accordingly, our results confirmed the hypothesis that CYP2E1 expression in 1,2-DCE-poisoned mice could be upregulated along with the exposure days and could be suppressed by DAS pretreatment. Studies by other researchers also demonstrated that expression of CYP2E1 could be depressed effectively at the transcriptional level by DAS treatment (Wargovich, 2006; Jin et al., 2013), which was consistent with the results of this study.

However, as reported in our previous study (Wang et al., 2014), exposure to 1.2 g/m^3^ 1,2-DCE for 3.5 h per day up to 3 days might cause brain edema, which was supported in this study by the increased water contents and typical morphological changes in the brains of 1,2-DCE-poisoned mice. Furthermore, our results also revealed that while the brain edema was formed, the contents of NPSH decreased, whereas MDA levels increased markedly in the brains of 1,2-DCE-poisoned mice. MDA is a marker of lipid peroxides resulting from ROS-induced cell membrane damage, reflecting the status of oxidative damage in tissues. NPSH is the main non-enzymatic antioxidant in cells and acts as a scavenger of oxidants through both enzymatic and non-enzymatic pathways. Therefore, our results suggested that oxidative damage might be induced in the brains of 1,2-DCE-poisoned mice. However, our results found that, in response to the suppressed expression of CYP2E1 in the intervention groups, the contents of NPSH in the brain increased, whereas MDA levels and water contents decreased markedly. Moreover, the pathological changes of brain edema were also improved via pretreatment of 1,2-DCE-poisoned mice with DAS. Taken together, the present results clearly demonstrated that CYP2E1-mediated metabolism of 1,2-DCE might contribute to oxidative damage in the brain, which, at least in part, underlies the mechanism of 1,2-DCE-induced brain edema. Similar results were also reported by Huang et al. (2002), in which both GSH levels and SOD activity in the brains of 1,2-DCE-exposed animals decreased and MDA contents increased. The results of our previous study showed that NPSH contents and SOD activity in the livers of 1,2-DCE-poisoned mice decreased and MDA levels increased; in contrast, NPSH levels and SOD activity in the livers of DAS-pretreated mice increased, while MDA levels decreased (Sun et al., 2016). Nevertheless, this is the first report to show the association between CYP2E1 expression and oxidative damage in the brain and their contributions to brain edema formation in 1,2-DCE-poisoned mice.

In addition, the above results were also supported by the changes in both Nrf2 and HO-1 expression in the brains of 1,2-DCE-poisoned mice. It is well-known that Nrf2 is an essential transcription factor in regulating the expression of antioxidant enzyme genes, and HO-1 is one of the antioxidant enzymes modulated by Nrf2 (Reichard et al., 2007). It has been reported that Nrf2 can be activated by ROS and can, in turn, promote the gene transcription of antioxidant enzymes (Gerjevic et al., 2011; Araujo et al., 2012). Therefore, the expression levels of both Nrf2 and HO-1 can be used as the indicators of intracellular oxidative stress (Chen Z. et al., 2015). This study found that the expression levels of both Nrf2 and HO-1 in the brains of 1,2-DCE-poisoned mice were transcriptionally upregulated and that DAS pretreatment could dose-dependently suppress the enhanced expression of Nrf2 and HO-1 in 1,2-DCE-poisoned mice. It has been documented that CYP2E1 can produce high levels of ROS during the metabolic process due to its high NADPH oxidase activity. Thus, it can be speculated that 1,2-DCE metabolism mediated by overexpressed CYP2E1 could produce excessive ROS in the brain, which might attack biological molecules to induce oxidative damage; on the other hand, ROS might also initiate the antioxidant defense system via activating Nrf2 to produce large amounts of antioxidants, such as HO-1, against the oxidative damage. Since DAS pretreatment could suppress the expression of CYP2E1 in 1,2-DCE-poisoned mice, and in turn reduce the production of ROS, as a consequence, the expression levels of Nrf2 and HO-1 dropped dramatically in the intervention groups. To the best of our knowledge, there have been few reported studies about the changes in Nrf2 expression in 1,2-DCE-poisoned animals.

The BBB plays a crucial role in maintaining stability in the brain, and the tight junctions between adjacent epithelial cells constitute the basic structure of the BBB to limit paracellular permeability (Liu et al., 2012). ZO-1 is a cytoplasmic accessory protein that binds to actin and associates with the cytoplasmic domains of occludin and claudins. Both occludin and claudins are integral membrane proteins, the extracellular loops of which originate from neighboring epithelial cells to form the paracellular barrier of tight junctions. ZO-1, occludin and claudins are the main components of tight junctions and are necessary for maintaining BBB integrity and permeability (Sandoval and Witt, 2008; Abbott et al., 2010). Disruption of tight junction proteins could increase BBB permeability, contributing to brain edema formation (Alves, 2014). The findings from this study demonstrated that the protein levels of ZO-1, occludin and claudin-5 decreased markedly in the brains of 1,2-DCE-poisoned mice, and DAS pretreatment could attenuate the decrease of tight junction proteins in 1,2-DCE-poisoned mice. These results were consistent with the pathological changes in the brain, suggesting that disruption of tight junction proteins in the BBB might be involved in brain edema formation. Moreover, combined with the changes of tight junction proteins in the intervention groups, it has been proposed that ROS and reactive metabolites generated in the CYP2E1-mediated metabolism of 1,2-DCE might be involved in the disruption of tight junction proteins. In our previous study, it was reported that the expression levels of both ZO-1 and occludin decreased markedly at the early phase of brain edema, induced by subacute poisoning with 1,2-DCE (Wang et al., 2018), consistent with present findings. In the current study, for the first time, we report the changes of claudin-5 expression in the brains of 1,2-DCE-poisoned mice and the association between the metabolism of 1,2-DCE and the disruption of tight junction proteins in the BBB.

Forelimb flexure is a typical syndrome reported by Bederson et al. (1986) in rats with infarction in greater than 20% of coronal sections. When normal rats are suspended one meter above the floor, held gently by their tails, they typically extend both forelimbs toward the floor. However, when the rats with infarction were suspended, they may flex their forelimbs contralateral to the injured hemisphere; all rats with limb flexure had histologically documented infarctions. Thus, forelimb flexion could be used to rapidly and accurately evaluate the cerebral injury. In this studies, it was observed and used as a syndrome to represent brain lesion in 1,2-DCE-poisoned mice, which provided further evidence supporting the role of CYP2E1-mediated 1,2-DCE metabolism in brain edema formation as mentioned above.

In the present study, the body weights of 1,2-DCE-intoxicated mice decreased markedly, and pretreatment with a CYP2E1 inhibitor rescued the loss of body weight in mice. As we reported previously, the mice exposed to 1.2 g/m^3^ of 1,2-DCE for more than 2 days displayed body tremors and forelimb flexure due to brain injury. Therefore, the voluntary exercise levels and consumption amounts decreased substantially among these mice, which might contribute to their loss of body weight. In contrast, because pretreatment of mice with a CYP2E1 inhibitor ameliorated 1,2-DCE-induced brain injury by suppressing the metabolism of 1,2-DCE, the voluntary exercise and feeding amounts of mice in the intervention groups were obviously improved.

In summary, our results suggested that CYP2E1 expression could be transcriptionally upregulated in 1,2-DCE-poisoned mice, which might enhance 1,2-DCE metabolism *in vivo* and induce oxidative damage and tight junction disruption in the brain, leading to brain edema. In this study, we could not confirm the role of brain CYP2E1 expression in brain edema formation in 1,2-DCE-poisoned mice since pretreatment with DAS could suppress the expression of CYP2E1 in both liver and brain. However, it has been suggested that both chloroacetaldehyde and ROS derived from the metabolism of 1,2-DCE in the liver cannot across the BBB into the brain due to their high bioactivity and the high aldehyde dehydrogenase activity at the BBB (Zhong et al., 2012). It has been well documented that both catalase and CYP2E1 are the key enzymes of ethanol oxidation in the brains of rodents. In contrast, it is well-known that alcohol dehydrogenase (ADH) plays a crucial role in ethanol oxidation in the livers of both rodents and humans. Studies reported by Zimatkin et al. (2006) indicated that catalase inhibitors and CYP2E1 inhibitors significantly reduced the accumulation of ethanol-derived acetaldehyde and acetate in brain homogenates. An ADH inhibitor significantly decreases acetate but not the acetaldehyde accumulation in the brain. Ethanol-derived acetaldehyde accumulation in the brains of acatalasemic mice (i.e., mice with a genetic catalase deficiency) was 47% of the control value and 24% in double mutants (i.e., mice with deficiencies in both catalase and CYP2E1). Therefore, it is reasonable to hypothesize that metabolism of 1,2-DCE inside the brain should be associated with 1,2-DCE-induced brain lesion.

## Author Contributions

XJ designed, performed, and interpreted the experiments and wrote the manuscript. YL, XT, GW, and FZ edited the manuscript. YJ conceived the study, designed and interpreted the experiments, and revised the manuscript.

## Conflict of Interest Statement

The authors declare that the research was conducted in the absence of any commercial or financial relationships that could be construed as a potential conflict of interest.
